# Integrated blood and organ profile analysis to evaluate ameliorative effects of kaempferol on 5-fluorouracil-induced toxicity

**DOI:** 10.1038/s41598-024-52915-6

**Published:** 2024-01-29

**Authors:** Abhilasha Sharma, Mehul R. Chorawala, Rakesh M. Rawal, Neeta Shrivastava

**Affiliations:** 1 Department of Life Science, University School of Sciences, Gujarat University, Ahmedabad, 380009 Gujarat India; 2 L.M. College of Pharmacy, Ahmedabad, Gujarat India; 3 Shri B.V. Patel Education Trust, Ahmedabad, Gujarat India

**Keywords:** Gastrointestinal cancer, Drug discovery

## Abstract

Colorectal cancer (CRC) treatment strategies encompass a triad of medical interventions: surgery, radiotherapy, and chemotherapy. Among these, the use of chemotherapy, specifically 5-fluorouracil (5-FU), has become a cornerstone in CRC management. However, it is imperative to explore novel approaches that harness the synergistic potential of chemotherapy agents alongside adjunctive compounds to mitigate the severe adverse effects that often accompany treatment. In light of this pressing need, this study focuses on evaluating Kaempferol (KMP) in combination with 5-FU in a DMH-induced CRC animal model, scrutinizing its impact on haematological indices, organ health, and gastrointestinal, hepatotoxic, and nephrotoxic effects. Remarkably, KMP demonstrated haemato-protective attributes and exerted an immunomodulatory influence, effectively counteracting 5-FU-induced damage. Furthermore, organ assessments affirm the safety profile of the combined treatments while suggesting KMP's potential role in preserving the structural integrity of the intestine, and spleen. Histopathological assessments unveiled KMP's capacity to ameliorate liver injury and mitigate CRC-induced renal impairment. These multifaceted findings underscore KMP's candidacy as a promising adjunctive therapeutic option for CRC, underlining the pivotal need for personalized therapeutic strategies that concurrently optimize treatment efficacy and safeguard organ health. KMP holds tremendous promise in elevating the paradigm of CRC management.

## Introduction

Colorectal cancer (CRC) is a common malignancy with a high global incidence and is one of the main causes of cancer-related mortality. The five-year survival statistics for early-stage disease, especially stage I or II, are rather optimistic, at 91% and 82%, respectively. However, if cancer has metastasized, the prognosis becomes extremely poor, with only a 12% survival rate for individuals with advanced-stage disease^1^. In the past few decades, numerous chemotherapeutics agents have gained approval for clinical use, including 5-Fluorouracil (5-FU), oxaliplatin, cetuximab, capecitabine, irinotecan, bevacizumab, etc.^2–4^. However, it's important to have a better understanding of side effects and patient management in order to reap the full benefits of new combination chemotherapy regimens.

The major backbone drug in systemic chemotherapy for CRC is 5-FU^5^. 5-FU metabolically transforms to its active form, 5-fluoro-deoxyuridine-monophosphate, in tissues and acts as a thymidylate synthase (TS) inhibitor^4^. Despite the significant therapeutic potential of 5-FU, its clinical use presents certain challenges^6–8^. One such concern is its hydrophilic nature, which causes rapid elimination from the body, limiting the duration of its activity and potential efficacy. Furthermore, 5-FU has a confined therapeutic dosage range, and its effects vary greatly between people, making appropriate dosing challenging and presumably contributing to increased toxicity to normal tissues in certain patients which eventually limits its clinical use. Mainly liver and kidney are involved in 5-FU metabolism and elimination from the body which eventually results in hepatotoxicity and renal toxicity during the metabolism process^9–13^. Beyond the liver and kidney, 5-FU has been shown to be detrimental to other tissues and organs. One of the most prevalent side effects of 5-FU is myelosuppression, which occurs when the drug inhibits the synthesis of blood cells, increasing the risk of anaemia, neutropenia, and thrombocytopenia^14^. This myelosuppression can result in an immune system that is impaired and an increased susceptibility to infections. Mucositis, diarrhoea, and stomatitis are other common adverse effects of 5-FU-induced gastrointestinal toxicity, which decreases the patient's quality of life^15–17^. Consequently, there is a pressing need to identify novel therapeutic agents that can be used in combination with 5-FU for the treatment of CRC. Such initiatives attempt to increase patient survival rates while also minimizing the negative side effects of the therapy.

As of now, there is no clinical drug that can prevent or cure 5-FU-induced toxicity in humans. Recent scientific interest has focused on the use of herbal and natural compounds, or their derivatives, to mitigate the adverse effects of chemotherapy (chemoprotectors)^18^. Kaempferol (KMP), a natural flavonoid, has been amply found in vegetables and fruits including onions, tomatoes, beans, cabbage, broccoli, apples, strawberries, grapes, and citrus fruits^18–21^. Due to the presence of a phenolic molecule in its structure, KMP has been shown to offer several therapeutic benefits, including antioxidant action^18,21^. Several bodies of evidence have underlined the anti-apoptotic, anti-neoplastic, anti-chemoresistant, anti-inflammatory, and antioxidative potential of KMP but none of them comprehensively investigate its curative effect in an animal model of CRC to assess its impact as a safe therapeutic agent.

Based on our prior investigations, we have established that kaempferol offers nephroprotective properties against renal damage induced by cisplatin^22^ and exhibits anti-neoplastic effects against colorectal cancer (CRC) and Triple-Negative Breast Cancer (TNBC)^23–25^. Building on this knowledge, the present study aims to delve deeper into the potential synergistic enhancement of the chemo-protective effects of 5-FU when combined with kaempferol in a DMH-induced CRC rat model.

## Material and methods

### Drugs and chemicals

The following drugs and chemicals were used in this study: 1,2-dimethyl hydrazine (DMH) Dihydrochloride (TCI Chemicals, Japan), 5-Fluorouracil (Sigma-Aldrich), and Kaempferol (Natural Remedies Pvt Ltd, India).

### Animals

Adult male Wistar rats (300 ± 30 g) were used to carry out the present study. They were obtained from the animal house facility at the L M College of Pharmacy, Ahmedabad, Gujarat, India. All animals were maintained in vented cages at a regulated ambient temperature of 22 + 2 °C, with a relative humidity of 60–70% and a normal light/dark (12 h/12 h) cycle, with unrestricted access to commercially available normal pellet food (Keval feeds, Vadodara, India). The Institutional Animal Ethics Committee (IAEC) approved the study's experimental procedure under the reference number LMCP/IAEC/22/0045. The study was carried out in compliance with the requirements of the "Committee for the Purposes of Control and Supervision of Experiments on Animals (CPCSEA)" of the Ministry of Fisheries, Animal Husbandry, and Dairying, Government of India and the ARRIVE guidelines. Efforts were made to diminution the distress of rats during experiments.

### Experiment protocol and treatment

After 1 week of acclimatization, rats were randomly divided into the following groups for a study period of 15 weeks (10 weeks of induction phase followed by 2 weeks of incubation period and 3 weeks of treatment phase):

*Group 1: 0.9% Saline group:* 1 mM EDTA-saline once a week for 10 weeks + 0.9% Normal Saline (for 21 days).

*Group 2: 0.5% CMC group:* 1 mM EDTA-saline once a week for 10 weeks + 0.5% CMC (orally for 21 days).

*Group 3: DMH group:* DMH (50 mg/kg body weight) once weekly for 10 weeks.

*Group 4: 5-FU group:* DMH (50 mg/kg body weight) once weekly for 10 weeks + 5-Fluorouracil (50 mg/kg BW; once weekly for three weeks).

*Group 5: KMP group:* DMH (50 mg/kg body weight) once weekly for 10 weeks + kaempferol (50 mg/kg BW of orally daily for 21 days).

*Group 6: 5-FU(HD)/KMP:* DMH (50 mg/kg body weight) once weekly for 10 weeks + FU (50 mg/kg B.W.) intraperitoneally once weekly for 3 weeks) + kaempferol (50 mg/kg BW of orally daily for 21 days).

*Group 7: 5-FU(LD)/KMP:* DMH (50 mg/kg body weight) once weekly for 10 weeks + FU (25 mg/kg B.W.) intraperitoneally once weekly for 3 weeks) + kaempferol (50 mg/kg BW of orally daily for 21 days).

A day after the end of the 21 days of the treatment phase, blood samples were collected from each rat through *retro-orbital* artery bleeding using 2% isoflurane anesthesia in two different tubes (with or without EDTA) for the measurement of blood cell count and other biochemical assay. Rats were sacrificed humanely by the cervical decapitation method, lapartomized, and the selected vital organs (liver, kidney, spleen, lungs, brain, heart) were excised, weighed, and macroscopically examined.

### Survival analysis

Throughout the 21-day treatment phase, the survival of animals was closely monitored and recorded. The *Kaplan–Meier* technique was used to calculate the percentage of animal survival.

### Whole-blood analysis

To assess the overall health, blood collected in K2 EDTA-coated collection tubes was used to provide valuable information about different types and quantities of blood cells present in the blood of different treatment groups using the Dia-Count 60 Auto Hematology Analyzer.

### Biochemical assay

Blood collected was allowed to clot by leaving the tube undisturbed at room temperature for 2–4 h, followed by centrifugation at 10,000 rpm for about 10 min. The serum separated was collected and used to assess liver function tests (aspartate aminotransferase (AST), alanine aminotransferase (ALT), alkaline phosphatase (ALP), Total Bilirubin, Gamma-glutamyl transferase (GGT)) and renal function test (urea, blood urea nitrogen (BUN), serum creatinine, total protein).

### Measurement of food and water intake

The total food intake (g) was recorded for each group during the treatment phase and analyzed for the effect of the treatment.

### Histopathological analysis

After sacrifice, tissues of the liver, kidney, and intestine were prepared for histological and molecular analysis. The tissue samples were washed with ice-cold saline, followed by overnight fixation in 10% neutral buffered formalin. Later the fixed tissues were dehydrated in different grades (50–90%) of ethanol for 24 h and then embedded in paraffin wax to prepare the block. The blocks were then used to cut the thin sections (4–5 μm) using a microtome. To study the histopathological degeneration in the above-mentioned tissues, Hematoxylin–Eosin (H&E) reagent was used to stain the slides. The analysis primarily utilized magnifications of 10 × and 40 × to capture an overall view of tissue architecture, cellular organization, and general morphology. Higher magnifications, specifically up to 100 ×, were selectively employed for detailed scrutiny of specific regions and structures when necessary. Four animals per group were used and 5 slides per animal were prepared, and the mean of 3 fields was examined using a microscope (Magnus MLXi) with a digital camera (Sony) and Image J software was used for analysis.

### Statistical analysis

The collected data, including survival rates, blood cell counts, liver and renal function tests, food and water intake, and histopathological scores, were first tested for normality of distribution using the Shapiro–Wilk test. If the p-value was greater than 0.05, the data were considered to be normally distributed. For normally distributed data, comparisons between different groups of animals were made using one-way and two-way ANOVA. For the histopathological scores, which are ordinal data and not normally distributed, we used the non-parametric Kruskal–Wallis test followed by Dunn’s post hoc test for pairwise comparisons. These tests were chosen as they are suitable for comparing more than two groups and do not assume a normal distribution. All statistical analyses were performed using GraphPad Prism software version 8. The results are presented as Mean ± SEM. A p-value less than 0.05 was considered statistically significant.

## Result and discussion

### Colorectal cancer establishment in Wistar rats

**Figure 1:**
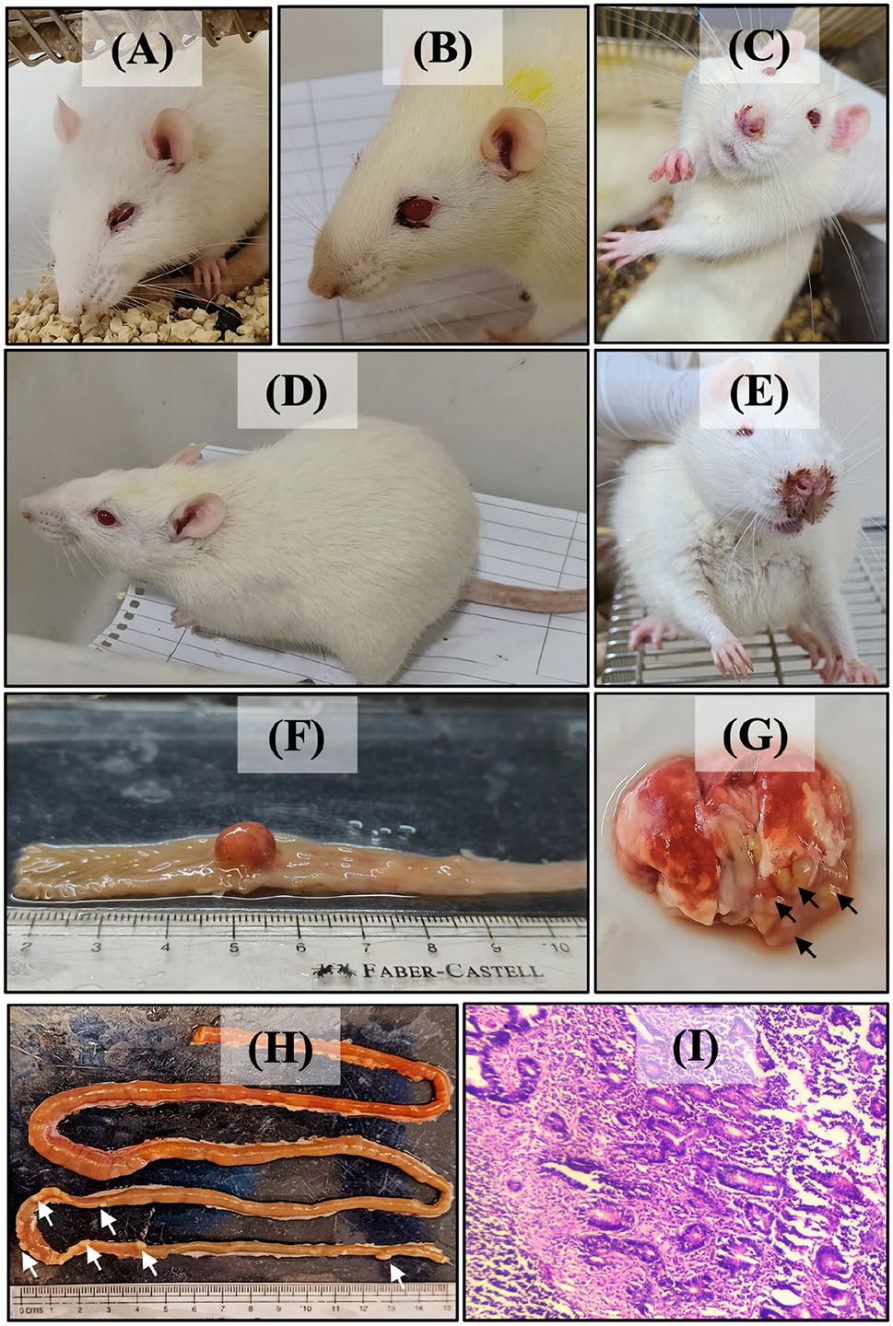
Successful establishment of DMH-induced colorectal cancer (CRC) in Wistar rat. Clinical Symptoms such as (**A**) Lethargy, (**B**) weakness, (**C**) running nose, (**D**) change in body position, (**E**) Chromodacryorrhea. Macroscopic analysis of (**F**) Colon, (**G**) Lungs and (**H**) small intestine of rats treated with DMH for 10 weeks. (**I**) Histopathological examination of colnic section using Hematoxylin and eosin stains at the light microscopic level (Scale bar 200 μm).

The DMH-induced CRC model is widely used in the experimental model due to its ability to replicate clinical symptoms, histopathological characteristics, and molecular changes as seen in human sporadic colon cancer^26,27^. In our study, we conducted a 10-week induction phase in rats, during which we observed several clinical symptoms, including lethargy, weakness, altered stool patterns, and changes in appetite (Fig. 1A–E). These symptoms were not evident in the normal rats (Group 1 and Group 2), indicating the successful induction of colorectal cancer-related symptoms in the experimental group.

At the end of the study, in the diseased control group (group 3) tumors were mainly located in the colon (Fig. 1F). However, tumors in the intestine and lungs were also found (Fig. 1G,H). Subsequent to tumor detection, tissue samples were extracted for in-depth pathological examination. The results of the histopathological analysis showed elevated tumor-infiltrating lymphocytes, indicating an immune response within the tumor microenvironment (Fig. 1I). Furthermore, we observed significant levels of necrosis within the tumor tissue, indicating widespread tissue cell death. The presence of severe necrosis, marked by tissue cell death, further underscored the aggressive nature of the tumor. Additionally, substantial disruption in tissue architecture was observed, underscoring the invasive characteristics of the tumor growth. This disruption suggests that the tumor cells were invading and infiltrating the surrounding healthy tissue, a hallmark of cancer progression.

Collectively, these findings provide strong validation that colorectal cancer was successfully established in rats. This successful establishment of colorectal cancer in rats sets the stage for further investigations into potential therapeutic interventions and assessments of treatment efficacy.

### Effect of KMP and/or5-FU on survival of animals

**Figure 2:**
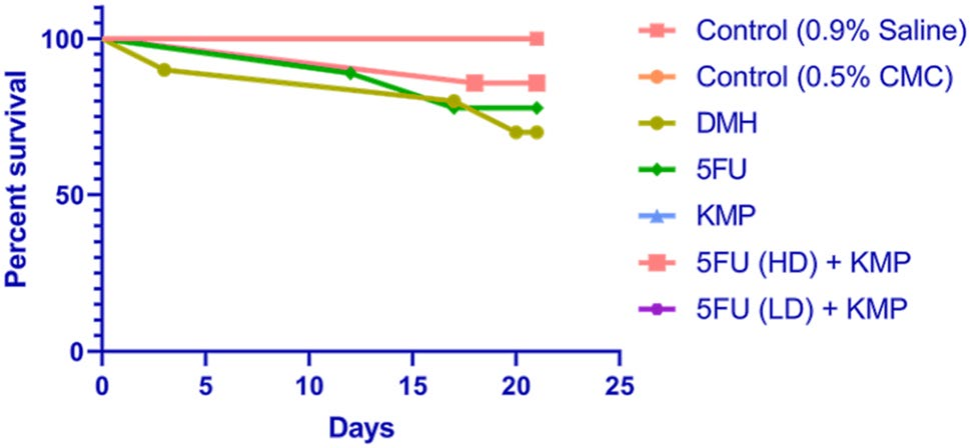
Effect of KMP and 5-FU on animal survival evaluated by *Kaplan–Meier* curve.

The survival analysis provides valuable insights into the impact of kaempferol (KMP) and 5-Fluorouracil (5-FU) in the context of colorectal cancer (CRC) induced by 1,2-dimethylhydrazine (DMH) (Fig. 2). Notably, the control groups (0.9% Saline and 0.5% CMC) demonstrated full survival, emphasizing the absence of adverse effects associated with the vehicle used in the experimental setup. The DMH group exhibited a lower survival rate of 62.5%, confirming the efficacy of DMH in inducing colorectal cancer and highlighting the severity of the model. On the other hand, the 5-FU group showed a 75% survival rate, suggesting its potential as a therapeutic intervention. However, it's essential to note that the survival rate did not reach 100%, indicating some limitations in the efficacy of 5-FU as a standalone treatment.

Interestingly, the DMH/KMP group demonstrated complete survival, indicating a protective effect of kaempferol against DMH-induced colorectal cancer. This aligns with previous findings in the study, showcasing the multifaceted benefits of KMP in maintaining organ integrity and countering the toxic effects of DMH.

The combination of 5-FU and KMP in both high-dose (HD) and low-dose (LD) scenarios demonstrated varying survival rates. The DMH/5-FU(HD)/KMP group showed an 87.5% survival rate, suggesting a positive impact of this combination. Meanwhile, the DMH/5-FU(LD)/KMP group exhibited complete survival, indicating a potentially synergistic effect in mitigating the adverse effects of DMH and 5-FU.

Collectively, the survival analysis corroborates the potential therapeutic benefits of kaempferol, particularly in combination with 5-Fluorouracil, in the context of colorectal cancer. The results underscore the importance of exploring synergistic approaches for enhanced efficacy and support the notion that kaempferol may play a pivotal role in improving overall survival outcomes in CRC treatment.

### Effect of KMP and/or 5-FU on haematological parameters

In this study, we conducted a comprehensive analysis of blood parameters among different treatment groups to gain valuable insights into the complex interplay between CRC induction, 5-FU chemotherapy, and the potential chemoprotective role of kaempferol (KMP). These findings shed light on the physiological consequences of CRC induction and its treatment, offering important implications for colorectal cancer management.

The increased monocyte level observed in the DMH-induced group strongly suggests the successful induction of CRC. Monocyte elevation is often associated with inflammation and immune responses triggered by cancer, affirming the effectiveness of our CRC induction model. This aligns with previous research^28–30^ that demonstrates the reliability of the DMH-induced CRC model in replicating clinical manifestations observed in human sporadic colon cancer. Table 1 shows the complete blood count of different groups. Specifically, animals in the CRC-induced group exhibited reduced red blood cell (RBC) counts, haemoglobin (Hb) levels, and platelet counts compared to normal control groups. These findings point to the development of anaemia and thrombocytopenia in CRC-induced rats, consistent with clinical observations in human colorectal cancer patients^28,31^. Anaemia can result from chronic bleeding, nutritional deficiencies, or the body's response to the tumor, while thrombocytopenia may be indicative of impaired bone marrow function often associated with cancer.

**Table 1:** Effect of KMP and/or 5-FU on haematological parameters.

Parameter	Control (0.9% Saline)	Control (0.5% CMC)	DMH	DMH/5-FU	DMH/KMP	DMH/5-FU(HD)/KMP	DMH/5-FU(LD)/KMP
Complete blood count							
RBC (mil/mm^3^)	9.7 ± 0.07	9.6 ± 0.09	7.6 ± 0.04	8.5 ± 0.06	10.1 ± 0.03	8.8 ± 0.06	8.1 ± 0.03
WBC (× 10^3^/mm^3^)	11.09 ± 0.74	11.15 ± 0.84	13.23 ± 0.71	7.29 ± 0.25^a,d^	8.16 ± 0.36^c,g^	10.41 ± 0.43^g,i^	8.61 ± 0.57^c^
Hb (g/dL)	15.35 ± 0.15	15.3 ± 0.1	12.25 ± 0.05^a^	14.05 ± 0.25	16.3 ± 0.1	15.05 ± 0.15	14.35 ± 0.85
Platelet (× 10^3^/μL)	1234 ± 384	1213 ± 0195	828.5 ± 59.5^b^	712.5 ± 12.5^a,d^	900.5 ± 83.5	1367.5 ± 46.5^a,h,i^	1248.5 ± 259.5^h,i^
Lymphocyte (%)	75.52 ± 3.2	75.85 ± 0.95	67.4 ± 2.4	76.35 ± 1.05	72.15 ± 0.05	65.7 ± 0.7^a,e,g,i^	69.35 ± 4.3^a^
Monocyte (%)	0.65 ± 0.05	0.6 ± 0.1	0.7 ± 0.1	2.6 ± 0.1	0.2 ± 0.3^c,e^	0.6 ± 0.1	0.7 ± 0.1^f.i^
Eosinophil (%)	0.85 ± 0.15	0.85 ± 0.05	2.35 ± 0.55^c^	0.45 ± 0.25	1.15 ± 0.15^a,f^	2.05 ± 0.05^a,f^	0.65 ± 0.45^d,f,i^
Neutrophil (%)	13.3 ± 0.06	13.05 ± 0.15	20.1 ± 0.08^a^	7.9 ± 0.25^c,e^	15 ± 0.07^a,d,f^	13.85 ± 0.25	11.85 ± 0.75^e,g^

The values are represented as Mean ± SEM for n = 6 animals/group.

^a^p < 0.001w.r.t control group, ^b^p < 0.01w.r.t control group, ^c^p < 0.05w.r.t control group, ^d^p < 0.001w.r.t DMH group, ^e^p < 0.01w.r.t DMH group, ^f^p < 0.001w.r.t DMH/5-FU group, ^g^p < 0.01w.r.t DMH/5-FU group, ^h^p < 0.05w.r.t DMH/5-FU group, ^i^p < 0.01 w.r.t DMH/KMP group, ^j^p < 0.05 w.r.t DMH/KMP group.

Interestingly, Co-administering kaempferol (KMP) with 5-FU led to a restoration of crucial haematological parameters, including RBC counts, Hb levels, and platelet counts. This suggests KMP's potential haematoprotective role, possibly through mechanisms like enhancing erythropoiesis, providing antioxidant protection, and reducing chemotherapy-induced haematological toxicity^32,33^. The precise mechanisms underlying this haemato-protective effect warrant further investigation. It is plausible that KMP may act on bone marrow progenitor cells, enhancing erythropoiesis and thrombopoiesis. Additionally, KMP might exert antioxidant properties, safeguarding blood components from oxidative damage induced by chemotherapy. These mechanisms collectively contribute to the amelioration of chemotherapy-induced haematological toxicity. Furthermore, our study revealed another facet of KMP's potential in the context of cancer treatment—its immunomodulatory properties. KMP appeared to counteract the suppressive effect of 5-FU on lymphocyte percentages. This finding underscores KMP's role in immune modulation^34^, which is of utmost significance in cancer therapy.

### Effects and safety of KMP on vital organs

**Figure 3:**
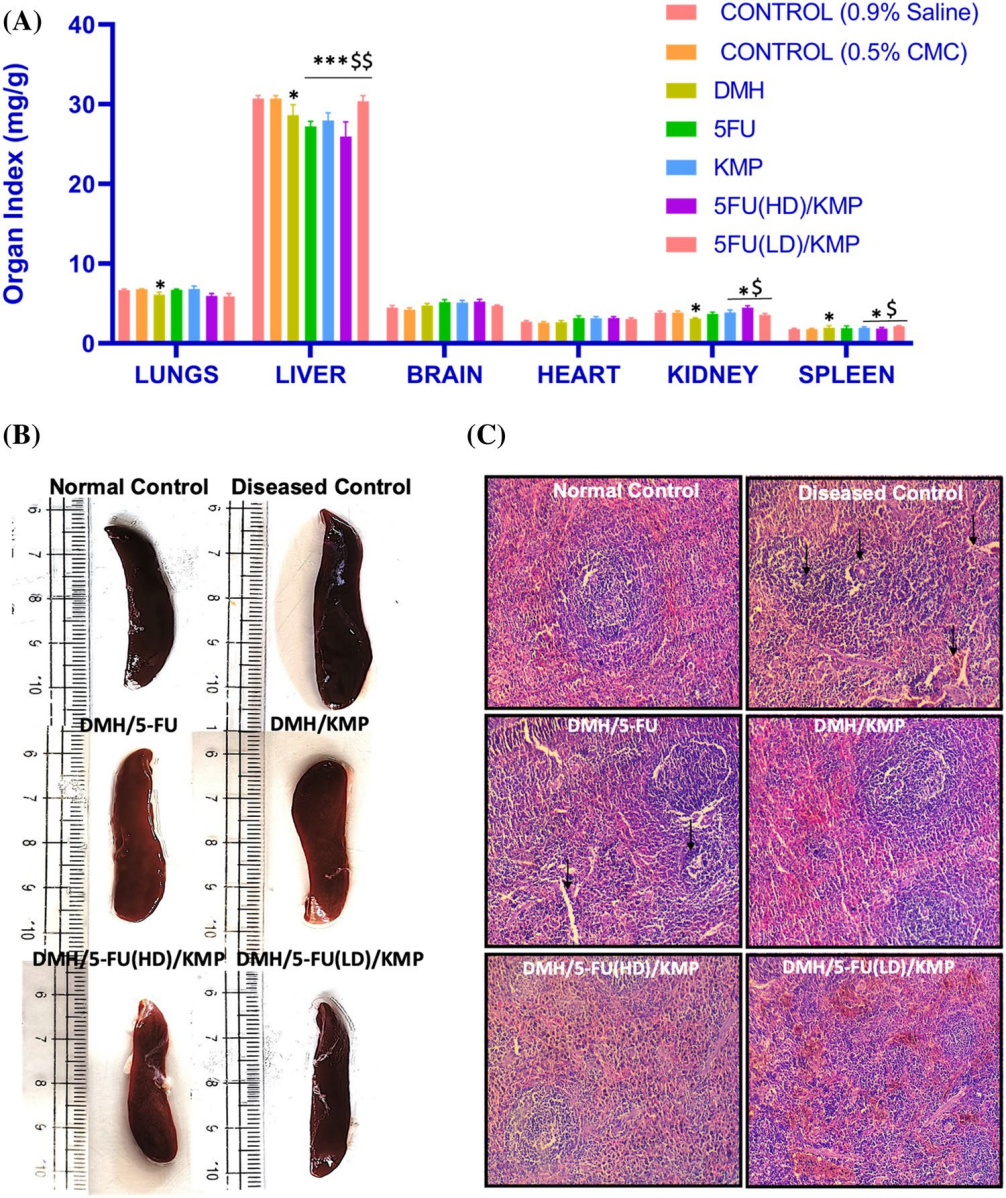
(**A**) Relative organ weights of rat organs (Lungs, Liver, brain, heart, kidney, spleen) in different treatment groups represented as Organ Index. (**B**) Gross examination of the effect of treatment on spleen length. (**C**) Histopathological examination of the spleen sections using Hematoxylin and eosin stains at the light microscopic level. A microscopic representative sample of groups: normal control; diseased control; treatment groups: 5-FU (50 mg/kg); KMP (50 mg/kg): 5FU (HD) (50 mg/kg) + KMP (50 mg/kg); 5FU (LD) (25 mg/kg) + KMP (50 mg/kg) (Magnification 10 ×) (Scale bar = 200 μm). → represents evidence of inflammation and fibrosis.

To get insight into possible structural or physiological impacts, organ indices are examined to see whether treatments or interventions result in changes in organ sizes. This technique aids researchers in determining how pharmacological treatments and other variables affect organ function and overall health. Organ Index is typically defined as the ratio of each animal’s organ weight to the total body weight of the animal^35,36^. Our study results confirm that no significant changes occurred in the size of organs including the brain, heart, and lungs of the treatment groups, and thus can be considered safe for CRC treatment (Fig. 3A).

Additionally, the examination of spleen length, coupled with histopathological alterations serves as a multidimensional approach to study treatment impact on splenic architecture. Our results show that control groups demonstrated consistent spleen lengths, reflecting baseline conditions (Fig. 3B). Enlarged spleen size was observed in the diseased control group, accompanied by increased cellularity and concomitant evidence of inflammatory infiltrates and fibrosis. Treatment with 5-FU displayed a restorative effect, characterized by partial regression in spleen length although sustained cellularity and inflammation were observed. Notably, the group treated with KMP either alone or in combination with 5-FU exhibited diminished spleen size and attenuated signs of inflammation and fibrosis (Fig. 3C). This implies the potential protective effects of KMP supplementation on ameliorating adverse effects on the spleen.

However, the liver index displayed a slight decrease in the diseased control and 5-FU administered group, implying potential damage to liver tissues. Similarly, minor differences among treatment groups display variation in kidney index. Therefore, to comprehensively understand the ramifications of the treatments on the liver and kidneys, further biochemical assays and histopathological analyses were performed.

### Effects of KMP on 5-FU-induced gastrointestinal mucosal damage

In our study, we meticulously tracked food and water consumption patterns across various treatment groups to gain deeper insights into the dietary and hydration behaviours during the treatment phase (Table 2). The observed variations in food intake are of particular interest. In control groups, we noted slight increases in food consumption over time, a phenomenon that aligns with the known pattern of animals exhibiting heightened food intake as they acclimate to their environment. However, a striking contrast was observed in CRC-induced groups, with the most substantial reduction in food consumption noted in the DMH-induced group. This pronounced decrease may be attributed to the physiological consequences of CRC induction. Colorectal cancer can disrupt normal metabolic processes and compromise appetite due to factors such as inflammation, tumor-induced changes in gut physiology, and the body's response to tumor progression.

**Table 2:** Effect of KMP and/or 5-FU on food and water intake.

Groups	Food consumption (g)		Water consumption (ml)	
	Initial (prior treatment phase starts)	Final (end of treatment phase)	Initial (prior treatment phase starts)	Final (end of treatment phase)
	Mean ± SEM	Mean ± SEM	Mean ± SEM	Mean ± SEM
Control (0.9% saline)	26.8 ± 0.4	28.4 ± 0.5	22.8 ± 0.3	24.4 ± 0.4
Control (0.5% CMC)	27.1 ± 0.3	29.1 ± 0.3	22.3 ± 0.1	24.7 ± 0.3
DMH	11.6 ± 0.2	12.4 ± 0.2^a^	17.4 ± 0.2	22.2 ± 0.1^a^
DMH/5-FU	9.8 ± 0.1	11.6 ± 0.4^a,b^	15.9 ± 0.2	20.4 ± 0.3^a,b^
DMH/KMP	13.8 ± 0.2	21.6 ± 0.3^a,b,c^	18.1 ± 0.4	25.8 ± 0.4^a,b,c^
DMH/5-FU(HD)/KMP	10.8 ± 0.3	16.6 ± 0.4^a,b,c,d^	17.3 ± 0.3	24.4 ± 0.2 ^a,b,c^
DMH/5-FU(LD)/KMP	12.8 ± 0.3	20.6 ± 0.4^a,b,c^	17.6 ± 0.4	22.9 ± 0.7 ^a,b,d^

The values are represented as Mean ± SEM for n = 6 animals/group.

^a^p < 0.001w.r.t control group; ^b^p < 0.001w.r.t DMH group, ^c^p < 0.001w.r.t DMH/5-FU group; ^d^p < 0.01w.r.t DMH/KMP group.

Moreover, our investigation into water consumption patterns revealed notable variations in response to treatment. While control groups exhibited relatively stable water consumption, CRC-induced groups displayed distinct changes in hydration behaviour. Notably, the DMH/5-FU(HD)/KMP group exhibited increased water consumption over time. This heightened thirst could be a response to the combined effects of CRC induction, 5-FU chemotherapy, and KMP supplementation, collectively influencing hydration needs.

**Figure 4:**
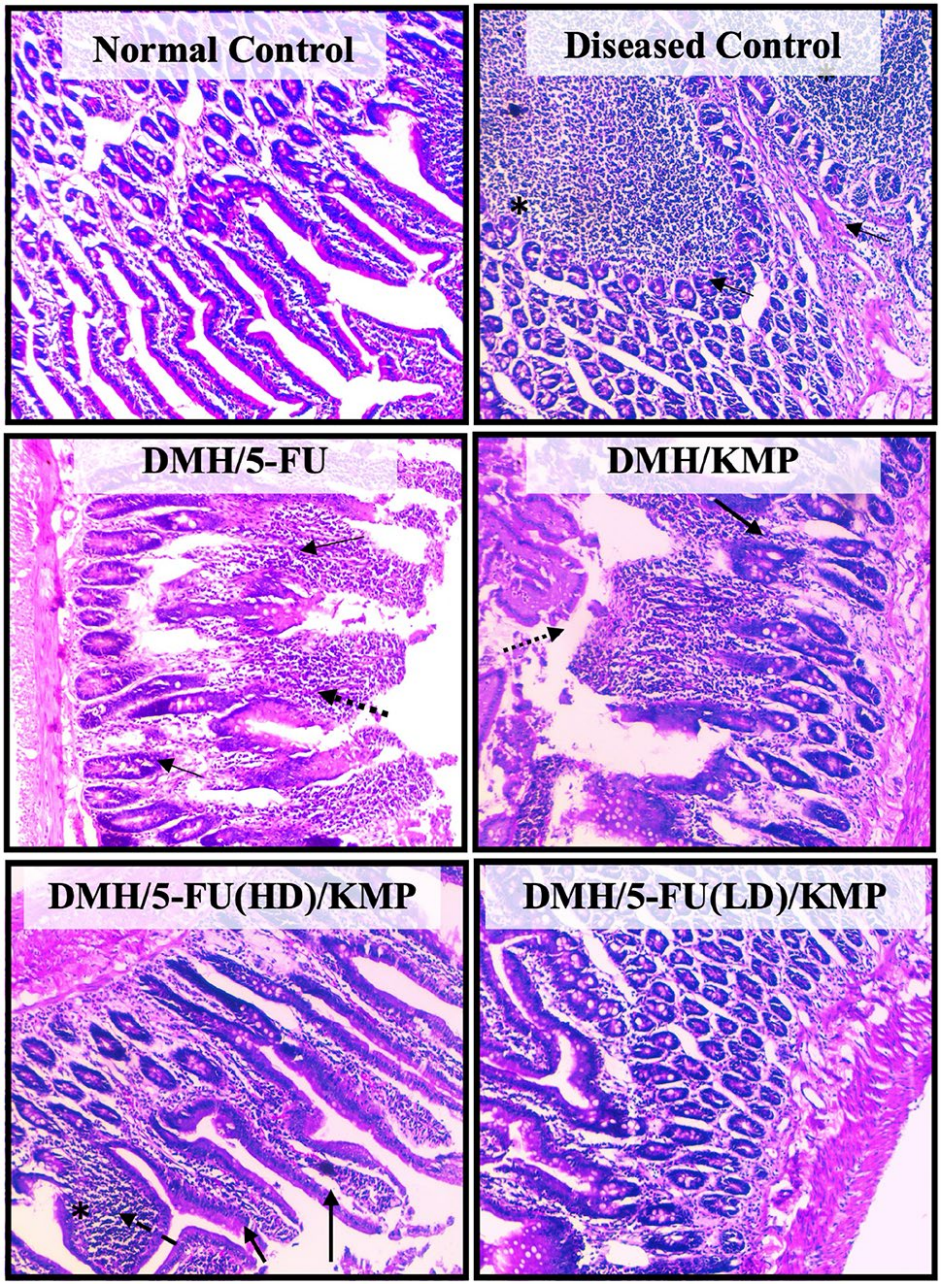
Histopathological examination of the small intestine sections using Hematoxylin and eosin stains at the light microscopic level. A microscopic representative sample of groups: Normal control; Diseased Control; treatment groups: 5-FU (50 mg/kg); KMP (50 mg/kg): 5FU (HD) (50 mg/kg) + KMP (50 mg/kg); 5FU (LD) (25 mg/kg) + KMP (50 mg/kg) (Magnification 10 ×) (Scale bar 200 μm). → represents infiltration of inflammatory cells in the lamina propia; *represents evidence of inflammation and necrosis; dotted arrow represents swollen crypts with infiltration of inflammatory cells.

To gain further insights into the structural and cellular alterations underlying these consumption patterns, we conducted histopathological examinations of duodenum tissue (Fig. 4). The results were illuminating. In the DMH-induced group, extensive damage, reduced cellularity, inflammation, and necrosis were evident in the duodenal tissues. These pathological changes likely contribute to decreased food intake as they can impair nutrient absorption and disrupt normal appetite regulation^37,38^. Conversely, the KMP group displayed different histopathological characteristics. Swollen crypts with inflammatory cell infiltration but no signs of necrosis were observed, suggesting that KMP supplementation might exert protective effects on the intestine. These effects may include mitigating inflammation and preserving tissue integrity, allowing for more efficient nutrient absorption and maintenance of food intake.

### Effects of KMP on 5-FU-induced hepatotoxicity

Our study rigorously assessed liver function through the analysis of key parameters, shedding light on the profound impact of colorectal cancer (CRC) induction and treatments, including kaempferol (KMP) supplementation, on hepatic health.

Total bilirubin and liver enzymes including SGPT/ALT, SGOT/AST, ALP, and GGT serve as crucial indicators of liver function (Table 3A). Our results reveal that control groups (0.9% Saline and 0.5% CMC) exhibited consistent bilirubin levels and liver enzyme levels, indicating normal hepatic function. In contrast, the CRC-induced group (DMH) demonstrated significantly reduced total bilirubin levels and elevated liver enzymes, potentially pointing to altered bilirubin metabolism linked to CRC. Strikingly, DMH/5-FU treatment further exacerbated this damage, underscoring the potential hepatotoxicity of 5-FU. However, the KMP group displayed a restoration of bilirubin levels, suggesting that KMP may have a hepatoprotective effect by ameliorating CRC-induced bilirubin abnormalities.

**Table 3:** Effect of KMP and/or 5-FU on liver function tests (A) and renal function tests (B).

Parameter	Control (0.9% Saline)	Control (0.5% CMC)	DMH	DMH/5-FU	DMH/KMP	DMH/5-FU(HD)/KMP	DMH/5-FU(LD)/KMP
(A) Liver function test							
Total bilirubin (mg/dL)	0.48 ± 0.02	0.48 ± 0.01	0.07 ± 0.01^a^	0.08 ± 0.02^a^	0.36 ± 0.02^e,f^	0.07 ± 0.01^a,h^	0.21 ± 0.05^b,d,g^
SGPT/ALT (U/L)	38.4 ± 0.76	37.11 ± 3.13	64.65 ± 1.64^b^	98.8 ± 1.28^c,e^	28.35 ± 0.03^a,d^	33.31 ± 5.34^b,g^	59.3 ± 5.48^b,g^
SGOT/AST (U/L)	98.3 ± 2.94	97.2 ± 1.15	165.5 ± 0.8^a^	205.85 ± 1.76^b,d^	67.9 ± 0.69^e,f^	109.93 ± 3.13 ^a,f,j^	123.95 ± 4.08^a,d,i^
ALP (U/L)	63.65 ± 0.25	63.2 ± 0.17	131.75 ± 25.72^c^	280.5 ± 23.07^a,e^	79.05 ± 6.26^c,e^	96.35 ± 8.13 ^d,f,i^	83 ± 18.87 ^b,d,e,i^
GGT (U/L)	2.55 ± 0.49	2.6 ± 0.41	8.25 ± 1.84^a^	9.6 ± 1.84^b^	5.45 ± 0.37^c,d,h^	3.2 ± 1.21^a,e,g^	7.05 ± 0.21^a,h^
(B) Renal function test							
Urea (mg/dL)	32.93 ± 1.11	32.75 ± 0.89	43.52 ± 2.01^a^	48.35 ± 0.86^b^	41.96 ± 0.82	40.72 ± 0.81	41.42 ± 0.69
BUN (mg/dL)	15.37 ± 0.52	15.41 ± 0.46	20.31 ± 1.41^a^	22.57 ± 1.61^a^	20.21 ± 0.75^a^	19.01 ± 0.38^a^	20.25 ± 0.86^a^
Serum creatinine (mg/dL)	0.42 ± 0.06	0.43 ± 0.01	0.65 ± 0.02	1.09 ± 0.28^a,d^	0.46 ± 0.05	0.7 ± 0.04^a,g^	0.65 ± 0.02

The values are represented as Mean ± SEM for n = 6 animals/ group.

^a^p < 0.001w.r.t control group, ^b^p < 0.01w.r.t control group, ^c^p < 0.05w.r.t control group, ^d^p < 0.001w.r.t DMH group, ^e^p < 0.01w.r.t DMH group, ^f^p < 0.001w.r.t DMH/5-FU group, ^g^p < 0.01w.r.t DMH/5-FU group, ^h^p < 0.05w.r.t DMH/5-FU group, ^i^p < 0.01 w.r.t DMH/KMP group, ^j^p < 0.05 w.r.t DMH/KMP group.

**Figure 5:**
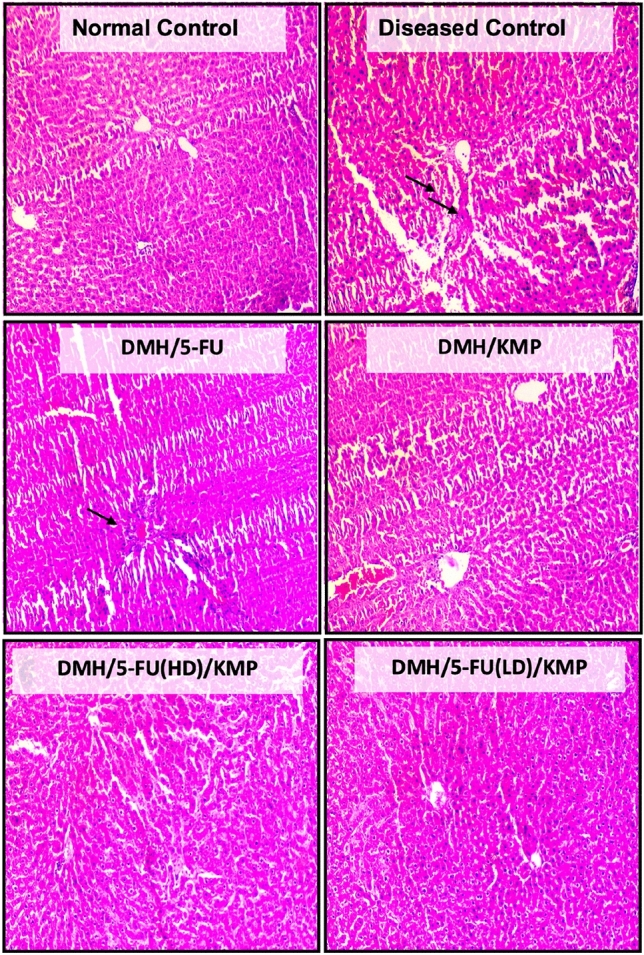
Histopathological examination of the Liver sections using Hematoxylin and eosin stains at the light microscopic level. A microscopic representative sample of groups: normal control; diseased control; treatment groups: 5-FU (50 mg/kg); KMP (50 mg/kg): 5FU (HD) (50 mg/kg) + KMP (50 mg/kg); 5FU (LD) (25 mg/kg) + KMP (50 mg/kg) (Magnification 10 ×) (Scale bar 200 μm) → represents the presence of inflammatory infiltrates or fibrotic changes.

Further, histopathological examination of liver tissues revealed distinct patterns among treatment groups: in the DMH-induced group, extensive liver damage, including distorted histology, cellular abnormalities, disrupted hepatocyte architecture, and potential inflammation or fibrosis, reflected the profound impact of CRC induction (Fig. 5). The DMH/5-FU group exhibited severe alterations in liver histology, marked by the loss of hepatocyte organization and significant tissue damage, aligning with elevated liver enzyme levels. Conversely, the DMH/KMP group displayed moderated cellular modifications, including disrupted hepatocyte arrangement, slight structural changes, and mild apoptotic cell presence, suggesting a protective role for KMP. Strikingly, the DMH/5-FU(HD)/KMP group exhibited severe liver histology disruption, confirming high-dose 5-FU's hepatotoxic effects, while the DMH/5-FU(LD)/KMP group showed limited inflammation without necrosis, suggesting a potentially less damaging response compared to the high-dose counterpart.

These findings align with existing literature on the hepatotoxicity of CRC and chemotherapeutic agents^39,40^. Colorectal cancer can profoundly impact liver function, leading to elevated liver enzymes and altered bilirubin metabolism. The exacerbation of hepatic damage by 5-FU is consistent with previous reports of its hepatotoxic effects^41,42^.

Notably, the potential hepatoprotective role of KMP, as indicated by restored bilirubin levels and milder histological changes, is in line with studies highlighting KMP's antioxidant and anti-inflammatory properties. Kaempferol has been shown to protect hepatocytes from oxidative stress and inflammation, which are key contributors to liver damage.

### Effects of KMP on 5-FU-induced renal damage

Our comprehensive study delved into renal function parameters and histopathological changes, unraveling the intricate interplay between colorectal cancer (CRC) induction, treatment strategies, and kidney health.

Control groups consistently maintained stable levels of urea, blood urea nitrogen (BUN), and serum creatinine, indicative of normal renal function (Table 3B). Conversely, in the CRC-induced group (DMH), elevated levels of these markers pointed towards significant kidney dysfunction, a finding in line with the observed histopathological evidence. Strikingly, treatment with 5-FU in the DMH/5-FU group exacerbated renal impairment, highlighting the nephrotoxicity associated with 5-FU chemotherapy. In stark contrast, the DMH/KMP group displayed a noteworthy restoration of renal function, suggesting the potential renoprotective effects of kaempferol (KMP), which merits further exploration.

**Figure 6:**
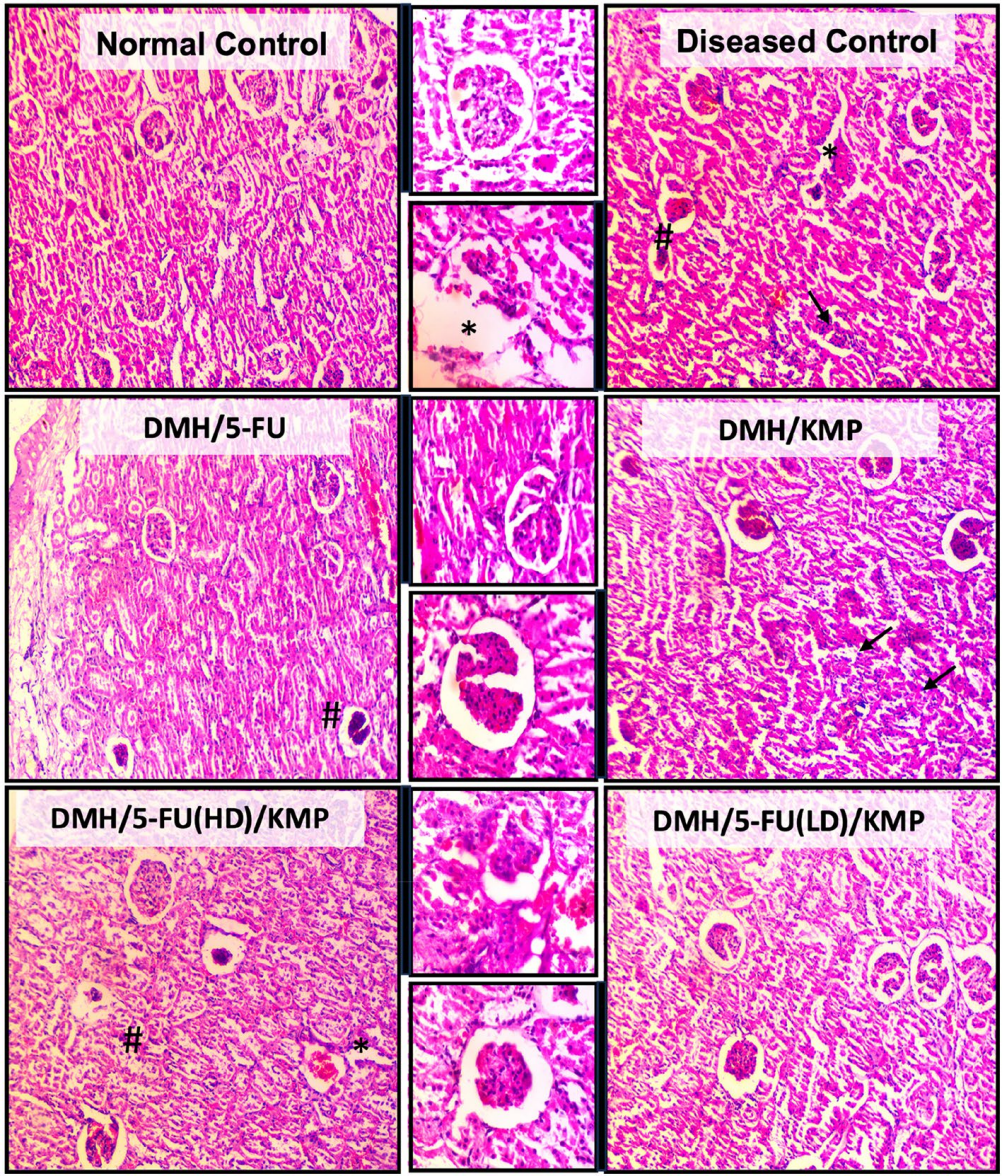
Histopathological examination of the kidney sections using Hematoxylin and eosin stains at the light microscopic level. A microscopic representative sample of groups: Normal control; Diseased Control; treatment groups: 5-FU (50 mg/kg); KMP (50 mg/kg): 5FU (HD) (50 mg/kg) + KMP (50 mg/kg); 5FU (LD) (25 mg/kg) + KMP (50 mg/kg) (magnification 10 × and 40 ×) (Scale bar 200 μm and 20 μm). → represents the significant lymphocyte infiltration; *represents tubular degeneration; ^#^represents degeneration of glomerular and congestion.

Histopathological examinations of renal tissues provided critical insights into the structural changes underlying these observed biochemical alterations (Fig. 6). In the DMH-induced group, pronounced glomerular degeneration, congestion, tubular degeneration, and substantial lymphocyte infiltration underscored severe kidney damage. The DMH/5-FU group displayed moderate glomerular degeneration and congestion, consistent with elevated renal parameters, thereby corroborating the adverse impact of 5-FU on renal function. Conversely, the DMH/KMP group exhibited increased infiltration of inflammatory cells, hinting at the protective role of KMP in kidney health. The DMH/5-FU(HD)/KMP group demonstrated mild glomerular degeneration, congestion, and tubular degeneration, implicating high-dose 5-FU treatment in renal damage^43^. Most intriguingly, the DMH/5-FU(LD)/KMP group showcased a normal renal morphology with no evidence of inflammation or necrosis, thereby highlighting a potentially less damaging response compared to the high-dose regimen.

Thus, our study underscores the imperative of considering renal health in the holistic management of CRC. KMP emerges as a promising candidate in attenuating renal damage, underscoring the pivotal role of personalized therapeutic approaches that prioritize both cancer treatment efficacy and organ health preservation.

Collectively, this study has the potential to advance the development of dietary supplements targeted at mitigating chemotherapeutic drug side effects and may offer valuable guidance for CRC patients seeking to ameliorate their condition through dietary interventions. However, it is important to recognize the intricate and multifaceted nature of human CRC risk factors. Consequently, further clinical investigations are imperative to elucidate and validate the favourable influence of Kaempferol (KMP) on CRC within the human population.

## Conclusion

In conclusion, our study has unveiled promising insights into the potential of kaempferol (KMP) as an adjunctive therapeutic option for colorectal cancer (CRC). The comprehensive investigation conducted in a CRC rat model demonstrated that KMP, in combination with 5-fluorouracil (5-FU), holds multifaceted benefits. KMP exhibited haematoprotective qualities, ameliorating 5-FU-induced haematological disturbances. It also showcased immunomodulatory effects and helped preserve the integrity of critical organs, particularly the spleen. Notably, KMP played a pivotal role in maintaining intestinal structural integrity and mitigating liver injury. The most striking discovery was KMP's renoprotective properties, which successfully countered CRC-induced kidney impairment. These findings underscore the potential of KMP to enhance the safety and efficacy of CRC treatment. As we advance toward personalized therapeutic approaches, our study emphasizes the importance of tailoring treatments to optimize CRC therapy while safeguarding the health of vital organs, including the liver. This research not only offers a promising avenue for future investigations but also highlights the potential for KMP to be a holistic and multifaceted asset in the battle against colorectal cancer, addressing various aspects of treatment-related challenges. In addition, future research should seamlessly integrate IHC, delving into the intricate molecular mechanisms that underlie KMP's therapeutic effects. This approach promises to elevate our comprehension, potentially unveiling additional dimensions of KMP's efficacy in the treatment of colorectal cancer.

## Data Availability

All data generated or analysed during this study are included in this published article. Additional raw data will be made available upon reasonable request to the corresponding author.
